# Barriers and facilitators to the implementation of nutrition standards for school food: a mixed-methods systematic review protocol

**DOI:** 10.12688/hrbopenres.13041.3

**Published:** 2021-05-13

**Authors:** Breda O'Mahony, Claire Kerins, Celine Murrin, Colette Kelly

**Affiliations:** 1 Home Economics Department, St. Angela's College, Sligo, Ireland; 2 Discipline of Health Promotion, National University of Ireland Galway, Galway, Ireland; 3 School of Public Health, Physiotherapy and Sports Science, University College Dublin, Dublin, Ireland; 4 Health Promotion Research Centre, School of Health Sciences, National University of Ireland Galway, Galway, Ireland

**Keywords:** Mixed methods, Systematic review, Barriers, Facilitators, School meal standards, School food guidelines, Implementation, Nutrition

## Abstract

**Background:** The importance of nutrition during childhood and the high prevalence of child and adolescent obesity has resulted in several countries implementing nutritional standards for school food as a way of providing healthy school food environments. Yet, there has been less focus on the barriers and facilitators influencing the process of implementing school food standards. This mixed-methods systematic review aims to address this evidence gap by synthesising the empirical evidence on the factors that may influence the implementation of school food standards.

**Methods:** This mixed-methods systematic review will use qualitative, quantitative and mixed-methods evidence from peer-reviewed publications retrieved from the following databases; PubMed, CINAHL, Scopus, EMBASE, Medline, PsycINFO and Web of Science. Grey literature will be accessed through Google Scholar, Open Access Theses and Dissertations, OpenGrey, RIAN, EThOS, ProQuest, WorldCat, Networked Digital Library of Theses and Dissertations, and public health organisation websites will also be accessed. Screening reference lists and citation chaining of all included studies will also be undertaken. No restrictions on publication date or language will be applied, however, only primary research studies relevant to supply-side stakeholders will be eligible for inclusion. Study quality will be assessed using the Mixed Methods Appraisal Tool. Study titles and abstracts will be screened to decide whether the full-text manuscript should be retrieved. For screening reliability, a second review author will assess a random sample of 20%. Kappa statistics will be used to assess inter-rater reliability, with values of 0.75 and higher representing high agreement. Two authors will independently extract data and factors reported to influence implementation. This will be synthesized using the Theoretical Domains Framework.

**Discussion:** A comprehensive understanding of these factors can provide guidance to relevant stakeholders to enhance the adoption, implementation and sustainability of nutrition standards for school meals.

**Systematic review registration**: PROSPERO CRD42019117904

## Abbreviations

MeSH, Medical Subject Headings; MMAT, Mixed Methods Appraisal Tool; PRISMA-P, Preferred Reporting Items for Systematic Reviews and Meta-Analysis Protocols; PROSPERO, International Prospective Register of Systematic Reviews; TDF, Theoretical Doman Framework; NSLP, National School Lunch Programme; V2, Version 2.

## Background

Schools are a key setting for the promotion of health and well-being ^1– 3^. They are one of the most effective ways of reaching a large segment of the population ^4, 5^, with no other institution having as much continuous contact and influence during the first stages of a child life ^6^.

One of the many ways that schools can support health is by the food that they provide ^7^. Good nutrition is associated with academic performance ^8^, psychological well-being and school attendance ^9, 10^. Up to a third of a child’s daily micronutrient intake can come from a school lunch ^11, 12^. Additionally, school meals can provide between 20 to 70% of a child’s energy requirements ^13^, thus further strengthening the need for healthy school meals. Coupled with this is a high prevalence of obesity among young people and the critical influence schools can play in supporting active living, healthy diets and body weight. This has resulted in many governmental school-based nutrition initiatives and policies, including nutrition standards for school meals being adopted ^13^. However, the effectiveness of school-based policies on childhood and adolescence nutrition and obesity depends on their implementation, which is often less than optimal, even when these policies are obligatory ^14^.

To date, a number of countries and regions around the world have introduced nutrition or food standards for school food on a mandatory basis. These include Finland in 1943 ^15^, Sweden in 1997 ^16^, Norway in 2001 ^17^, a reintroduction of compulsory guidelines after 21 years in England in 2001 ^18^, Slovenia in 2010 ^19^, and an updated National School Lunch Programme in the United States of America (USA) in 2012, which will be phased into all schools by 2023 ^20^. Nutrient standards are based on limits and promotions of various nutrients, whereas food-based standards set requirements on what food can and cannot be served and how frequently ^21^. Examples of standards include the Irish Nutrition Standards for School Meals ^22^. The food-based standards set out requirements for each meal type e.g. for breakfast, a minimum of two items should be provided, one serving of wholemeal or wholegrain cereals and breads, and one serving of either milk, yoghurt or cheese or fruit.

Differences exist in the provision of school food in Europe and internationally, and even from school to school within countries. Providing school food that meets nutritional guidelines or standards is complex ^16, 23^. Some countries provide school meals for all their students ^24^, regardless of their socio-economic environment ^25^, whilst in other jurisdictions the responsibility lies with the individual school ^24^. Other factors that contribute to the complex provision of school food include ensuring canteens make a profit ^26^ and organisational implications for principals ^27^. The latter includes existing contracts between food operators and schools that are agreed, based on the provision of catering infrastructure in schools ^27^.

It has been noted that more support must be provided to schools to allow the implementation of nutritional guidelines ^28^. Data from Gregoric and colleagues (2015) ^13^ found that the implementation of school nutrition guidelines differed by schools within the same region. Implementation as a process and a science is complex to study because of the numerous factors affecting implementation, its process, results and potential solutions ^29^.

Critical stakeholders involved in the implementation of food-based guidelines are supply-side stakeholders ^23, 30^ i.e. food service directors, catering managers and staff, school management, programme coordinators and contracted catering suppliers. Some of the factors related to implementation in schools include difficulties associated with preparing and serving fresh food at school ^20^; inadequate canteen facilities ^31– 33^; spending excessive time completing funding applications ^30^; and the requirement of staff training around the food guidelines ^20, 34, 35^. Positively, in contrast, caterers in the UK found the food standards relatively easy to achieve ^36^. However, there has been little synthesis of this research, particularly from the perspective of supply-side stakeholders.

Developing and improving strategies to increase supply-side stakeholders conformity of school meal standards requires a comprehensive understanding of the factors that enable and hinder implementation. One such framework that can allow us to apply theory to comprehensively identify factors that need to be addressed is the Theoretical Domains Framework (TDF) ^37^. The TDF was developed from 128 theoretical constructs from 33 theories that were perceived to be most relevant to implementation questions ^38, 39^. It was first published in 2005 ^40^ but later validated in 2012 (version 2 (v2)) ^41^. It has been used in numerous reviews to understand barriers and facilitators to a wide variety of behaviours ^41– 43^ and has confirmed validity and reliability ^44, 45^. Such reviews include implementation of dietary guidelines in early childhood education centres in Australia ^46^ and barriers and facilitators to the implementation of physical activity policies in schools ^47^. The framework (v2) provides 14 domains, which can capture a range of factors that influence implementation outcomes. These include knowledge, skills, memory, attention and decision processes, behavioural regulation, social/professional role and identity, beliefs about capabilities, optimism, beliefs about consequences, intentions, goals, reinforcement, emotion, environmental context and resources, and social influences ^48^.

A number of studies have identified various factors that influence implementation. Yet there is no published systematic review in this area that has adopted a bottom-up policy implementation perspective by focusing on the experiences and views of supply-side stakeholders, a key group in the adoption of these standards. Given this evidence gap, the primary aim of the systematic review is to collate the factors that influence the implementation of nutrition or food standards for school food provision in primary and post-primary settings (children aged 5–18 years). The use of the TDF to synthesise findings provides a mechanism to identify theoretical constructs to target in the development of food standards/guideline implementation. Using a theory provides a strong foundation for policy development, in contrast to simply identifying the barriers and facilitators ^49^. Understanding these factors from a theoretical perspective will provide a list of modifiable factors to target. This will help to inform future planning, improve uptake and practice of standards. Essentially, this review can guide policy-makers, researchers and individuals responsible for devising and implementing nutrition standards in schools.

## Review objectives

The primary objective is to identify and synthesise the existing evidence on the barriers and facilitators to implementing food or nutrition standards for school food from supply-side stakeholders. A secondary objective is to compare the barriers and facilitators between a primary and post-primary school setting.

## Methods

This mixed-methods systematic review is registered with the international database of prospective systematic review; Prospective Register of Systematic Reviews (PROSPERO): CRD42019117904 (25 ^th^ June 2019). The Preferred Reporting Items for Systematic Reviews and Meta-Analysis Protocols (PRISMA-P) checklist has been assessed in the preparation of this protocol (see *Reporting guidelines*) ^50^. The review will be conducted in accordance with PRISMA statement guidelines.

### Study eligibility criteria

The PICOS acronym (Population, Intervention, Comparison, Outcome and Study design) will be used to select study criteria, as described below. PICOS was selected due to achieving a comprehensive search with greater sensitivity than specificity ^51^.

***Population***. To be eligible for this review, studies have to include data which focuses on stakeholders who have a role in the implementation of nutrition or food standards or guidelines for school food within primary and post-primary school settings. Supply-side stakeholders refers but is not limited to catering management and staff, school principals/managers, contracted catering suppliers, food service directors and managers, and programme coordinators. It will also include studies that allude to officials from government organisations that may influence food provision in schools e.g. policy-makers. As this is an international review and to avoid differences that exist from country to country, e.g. age, all types of primary and post-primary schools will be included (Junior, Elementary, Middle, Secondary, Senior and High school). Standards in pre-schools, post-secondary schools and third-level settings will not be included. Furthermore, studies involving school children's perceived barriers and facilitators will also be excluded. Students are not included as they are classified as demand-side stakeholders.

***Intervention***. We will include studies of interventions delivered in educational establishments where the standards for school food have been implemented on a voluntary or mandatory basis. This includes food and nutrient standards for all meals, beverages and snacks provided in schools, including breakfast clubs and vending machines. As different jurisdictions implement different types of standards, both nutrient and food-based standards will be included. Studies on school nutrition policies and healthy eating interventions will not be included unless such policies and interventions are based on school meal standards. Similarly, studies on health promoting schools will not be included unless data specific to school-based standards can be extracted. The decision to include studies where voluntary school food standards were implemented is based on a preliminary scoping search.

***Control***. Whilst no comparator is being studied in this review, studies will not be excluded on the basis of having a comparator or control group.

***Outcome***. The primary outcome will include any barrier or facilitator to the implementation of nutrition and food-based standards for school food. For this review, we will use a similar definition that Kerins *et al.* applied in a systematic review protocol ^52^. A barrier is defined as any variable that impedes or obstructs the implementation of nutrition standards, whereas a facilitator is defined as any variable that eases and promotes the implementation of nutrition standards. The findings may include the following: (i) verbatim quotations from research participants; (ii) excerpts, quotations or entire passages from studies using documentary analysis; (iii) narrative descriptive summaries of results; and (iv) statistical analyses from surveys and questionnaires. A secondary outcome of the review is to compare the barriers and facilitators between a primary and post-primary school setting.

***Study design***. We are conducting a mixed-method systematic review; therefore quantitative, qualitative and mixed-method studies will be accessed. The rationale for this choice is to capture a comprehensive understanding of the factors that affect implementation. This may include, but is not limited to, the following studies which use appropriate methods of data collection and analysis (i) qualitative studies; case studies, grounded theory, ethnography, action research studies (ii) quantitative studies; case-control studies, quasi-experimental studies, randomised controlled trials, cross-sectional studies and (iii) mixed-methods (combining qualitative and quantitative methods of data collection and analysis); focus groups, interviews, surveys, questionnaires, observation. This review will disregard editorials, commentary and opinion pieces.

***Language***. There will be no restriction on language. This is to ensure all suitable international research on nutrient or food-based standards is captured.

***Publication year***. There will be no restriction on publication year.

### Search strategy

A search of peer-reviewed literature combining, where possible, published search filters for school meals, barriers or facilitators, will be undertaken. Guidance of an experienced librarian and discussion amongst the review team will also take place to inform the strategy. Broad search terms will be used to garner greater sensitivity than specificity so as to ensure a comprehensive search is undertaken ^53^. Databases relating to various fields, including education, food, and nutrition will be used. Each search strategy will be database specific and will include applicable elements such as Medical Subject Headings (MeSH) (or equivalent), truncation, Boolean operators and will be adapted where appropriate. Initial scoping searches will be undertaken by the lead review author to refine the search strategy. Table 1 illustrates a sample search strategy for the CINAHL database. The following electronic databases will be searched: PubMed, CINAHL, Scopus, EMBASE, Medline, PsycINFO and Web of Science. To identify published government reports and other grey literature, searches through Google Scholar, Open Access Theses and Dissertations, OpenGrey, RIAN, EThOS, ProQuest, WorldCat, Networked Digital Library of Theses and Dissertations, and public health organisation websites will also be undertaken. Furthermore, this minimises the influence of publication bias and produces a balanced picture of available evidence ^54^. To identify any additional studies, the reference lists of all included studies will be screened to retrieve additional eligible articles ^55^. All search results will be reviewed for eligibility, except in the case of Google Scholar where the first 200 citations will be screened. A priori decision to screen the first 200 hits on Google Scholar, as sorted by relevance, was decided after considering the time required to screen each hit ^53^. The lead or corresponding authors for all included studies will be contacted (via email with two attempts) so as to identify on-going or unpublished research studies that may be relevant to this review. To ensure that the search strategy is undertaken in a systematic way, a memoing method will be used to record the working notes when conducting preliminary searches as well as documenting the protocol-driven search strategy ^56^.

**Table 1.** Sample CINAHL title and abstract search strategy.

Search number	Search string
#1	recommend* OR guideline adherence [mh] OR guidance* OR protocol* OR nutrition policy [mh] OR strateg* OR standard* OR nutri* OR health promotion [mh]
#2	school lunch* OR school meal* OR canteen* OR food services [mh] OR school food OR menu planning [mh] OR food program* OR school* meal* program* OR school dinner*
#3	school* OR education*
#4	#1 AND #2 AND #3

Mh MeSH headings

### Study selection

***Data management***. EndNote X9 will be used to manage references throughout the review. Once the searches have been carried out, the search results will be exported to EndNote. This will identify any duplicates, which will then be removed.

***Screening***. Search results will be imported into an online systematic review software, Rayyan. This will enable screening, data extraction and quality assessment. This will be undertaken after a piloted, clear and detailed set of inclusion and exclusion criteria has been drawn up (see *Extended data*) ^50^. The lead author will screen study titles and abstracts to decide whether the full-text manuscript should be retrieved. For screening reliability, a second review author will assess a random sample of 20%. Kappa statistics will be used to assess inter-rater reliability, with values of 0.75 and higher representing high agreement ^57^. In the case of disagreement, full article will be screened. Each study will be categorised into (a) potentially meeting the eligibility criteria or (b) not meeting the eligibility criteria. For all potentially eligible studies, full-text manuscripts will be obtained. A full-text screening process will then commence by two independent reviewers, which will then produce a final set of papers to be included in the review. In situations where the study eligibility cannot be resolved via consensus, a third review author will be consulted. A flow diagram will be completed to record the numbers of papers through each stage of the search and screening process, as recommended by the PRISMA guidelines ^58^.

### Quality assessment/risk of bias

Quality appraisal will be conducted by two independent reviewers, using the Mixed Methods Appraisal Tool (MMAT) (2018 version). This assessment tool was selected as it is used to efficiently appraise the most common methods and methodologies i.e. qualitative, quantitative and mixed-methods studies, with few generic quality criteria ^59, 60^. Additionally, the tool was designed to appraise the methodological quality of studies in a mixed-methods systematic review and not the quality of report writing ^61^. The MMAT focuses on methodological criteria ^59^ and includes two screening questions and nineteen questions corresponding ^61^ to the following five categories of study design; qualitative research, randomised controlled trials, non-randomised studies, quantitative, observational descriptive, and for mixed-methods studies ^59, 62^. For each study type, reviewers will quality score using a MMAT table. When disagreements between reviewers cannot be easily resolved, a third independent reviewer will become involved in order to reach a consensus.

### Data extraction and synthesis

For data extraction and synthesis, we have adopted a similar approach undertaken in a study protocol Graham-Rowe and colleagues ^63^. Data will be extracted from all full-text studies that meet the inclusion criteria, using a data extraction form. The data extraction form will be created using Microsoft Word. Data to be extracted will include, but is not limited to, the following: Key study information which will include study title, name of the first author, year of publication, country of study, language, study type (qualitative, quantitative and mixed methods studies), study design (e.g. cross-sectional and observational), intervention type (e.g. food standards, nutrient standards), type of implementation (voluntary or mandatory participation), educational setting (primary, post-primary, academies etc.) and participant characteristics (canteen staff, head-teacher, contracted catering suppliers, food service directors and managers, programme coordinators etc.), sample size, data collection and analysis methods. Data on intervention effects/outcomes, such as change in children’s dietary habits will not form part of this review. A coding manual with the definitions outlined by Cane *et al*., for the 14 theoretical domains from the TDF, will also be prepared. 

### Analysis

***Step 1: Data extraction***

Two review authors, not blind to author or journal information will independently extract from all the full-text studies that fulfil the inclusion criteria, using a data extraction form. Each data point will be categorised as either (a) raw data (e.g. quotes from qualitative studies); (b) analysed data from results sections (e.g. statistical analysis from surveys and questionnaires (c) interpretative descriptions and summaries of results from published reports. For qualitative studies, both the theme and the original reporting format of the barrier or facilitator will be extracted. This will include participant quotes, excerpts or entire passages from studies using documentary analysis, and narrative descriptive summaries of results. Whereas in quantitative studies, the frequency of all the barriers and facilitators will be extracted. Where mixed-method studies are included, data will be extracted into the relevant qualitative and quantative extraction forms.

### Deductive analysis

***Step 2: Coding of extracted data***

   (a) Pilot of the coding of data

To ensure validity and reliability, a pilot be will undertaken to allow the review authors to practice coding extracted data into the TDF domains. This will enable comparison of data coding and implement modification, if required. This will be jointly undertaken by the two review authors using the domain definition manual. Any discrepancies will be resolved by the review team, and if necessary by a third reviewer.

   (b) Coding extracted data to the TDF

The lead author will continue to code the extracted data, using the domain definition manual, to the TDF domain that they judge to best represent the factor. If a reported barrier or facilitator is judged to represent more than one domain, it will be coded multiple times into its associated domains. Where a factor is not recorded as a barrier or facilitator, we will revert back to the definitions. This is important so as to be able to highlight the pertinent factors affecting implementation and provide relevant recommendations. To access coding reliability, a second review author will assess a random sample of 20%. Kappa statistics will be used again to assess inter-rater reliability, with values of 0.75 and higher representing high agreement.

### Inductive analysis

***Step 3: Thematic synthesis***

Inductive thematic synthesis, based on methods by Thomas & Harden ^64^ will also be used to code any data that does not fit into the TDF. This will require a three-step process. Step one involves line by line coding of the data that was not coded to the TDF. The second step involves organisation or grouping of these code into associated areas to construct descriptive themes. In step three, the descriptive themes will be compared to refine the relationship between them so as to generate analytical themes.

### Sensitivity analysis

Following the synthesis stage, the first author will perform a sensitivity analysis. This will determine if the review findings are sensitive to the following variables: study quality, study methodology (qualitative, quantitative or mixed methods), type of implementation (voluntary or mandatory participation) and location. Both bias and sensitivity reviews will help ensure quality, rigour and transparency ^65^

### Importance criterion

To determine which domains are likely to be the most important factors influencing implementation, each domain in step two will be reviewed. In this review, the frequency of each domain will be examined. Similar to a study protocol by Graham-Rose *et al*. ^63^, for qualitative studies we will then consider ‘expressed importance’ within each domain by looking for a statement(s) from the authors interpretation of the study findings articulating what beliefs were reported to be important by the study participants. We acknowledge that this is not a precise process but it has a good fit with the qualitative approach as the meaning, interpretation and prioritisation of the data will be by the authors who have closer familiarity with the primary data, than will be possible by the review team. This process will require discussion and critical judgement by the research team. This will allow the review team, to interpret the domains that have the highest frequency and ‘expressed importance’ as the most important factors in the implementation of school meals standards/guidelines.

If there is sufficient data, we will explore whether the domains identified as important vary according to the educational establishment i.e. primary and post-primary schools. This will be performed by subgroup analysis. Findings will be described in tabular format.

### Study status

This study has not yet commenced.

## Discussion

The internal school food environment is considered to have a significant influence on student’s food consumption ^66^. It is believed that over 35% of their energy is obtained at school ^67^. Moreover, in many instances a school meal may be the only complete meal that students have access to ^68^. In response to the need of schools to play a more supportive role in obesity prevention ^69^, many jurisdictions have implemented policies and practices, one of which is food or nutrition standards for school meals ^13, 70^.

It is believed that the UK has the most comprehensive set of nutritional standards for school meals. However, the implementation of these standards has not necessarily resulted in better consumption and nutritional outcomes ^71^. Therefore, it is important to evaluate the process, to aid the full implementation of nutrition standards. Implementation evaluation measures the results from a process ^72^ and enables the transformation of policy plans into action ^73^. However, there are many individual, environmental and socio-cultural factors that can affect the successful implementation of policies ^49^. This is particularly pertinent to schools which are complex, with numerous factors that can influence implementation ^74^, the quality of implementation and the expected outcomes of the policy ^75^. The use of the TDF provides a holistic approach as it considers the complex interaction of the how and why ^76^, which must be taken into account when considering how nutrition standards for school meals are implemented in school settings.

A number of studies have identified various factors that affect implementation, however, there has been limited synthesis of this information from a supply-side stakeholder’s perspective. Ronto and colleagues (2020) ^77^ recent systematic review analysed the implementation and/or compliance with school-based healthy food and beverage policies. The eligibility criteria included policy relating to nutrition guidelines, regulations and/or restrictions on food and beverage availability, advertisement, placement or price. There was no exclusion criteria in relation to study participants, and grey literature was not accessed. Our mixed-methods review is specific to the implementation of food/nutrient-based standards from a supply-side stakeholder’s perspective using a variety of electronic databases and grey literature sources. Given the potential impact that school meal guidelines and standards can have on the health and well-being outcomes of children and adolescents, understanding the factors that affect their implementation is key. We are confident that the depth of this review will provide a holistic understanding of the factors as all types of studies, qualitative and quantitative or both, including grey literature, will be accessed. Furthermore, there will be no language or publication date restrictions. The review will follow academic rigour and will include a number of strategies for validity, reliability and to reduce the effects of bias. This will be achieved by having clear and detailed inclusion and exclusion criteria, independent reviewers, the use of PRISMA guidelines, a MMAT, and by using computer packages for data and quality management. Finally, where deviations from this protocol occur, this will be justified and discussed in the systematic review upon publication and will be documented on PROSPERO.

The outcomes of this study will be applicable to policy-makers and their advisors, practitioners, researchers and school administrators responsible for supporting the implementation of nutrition standards. Documenting barriers is necessary to improve the implementation of policy changes ^49^. Furthermore, a theoretical based framework will be used, which will provide a greater insight into the complexities of implementation. It will also have the capacity to steer future developments and implementations.

When completed, the review results will be submitted for publication to a peer-reviewed journal with the potential of writing a policy brief targeted at key stakeholders. Where applicable and accepted, findings will be disseminated and communicated at conferences, workshops, seminars, and via social media.

## Data availability

### Underlying data

No underlying data are associated with this article.

### Extended data

Open Science Framework: Barriers and facilitators to the implementation of nutrition standards for school food: a mixed-methods systematic review, https://doi.org/10.17605/OSF.IO/6Q24P ^50^.

This project contains the following extended data:

- Supplementary File 2. Inclusion and Exclusion Criteria.pdf

### Reporting guidelines

Open Science Framework: PRISMA-P checklist for ‘Barriers and facilitators to the implementation of nutrition standards for school food: a mixed-methods systematic review’, https://doi.org/10.17605/OSF.IO/6Q24P ^50^.

Data are available under the terms of the Creative Commons Zero “No rights reserved” data waiver (CCO 1.0 Public domain dedication).
