# Supplementary material for: Barriers and facilitators to the implementation of nutrition standards for school food: a mixed-methods systematic review protocol
Source: HRB Open Res. 2021 May 13;3:20. Originally published 2020 May 4. [Version 3] doi: 10.12688/hrbopenres.13041.3 (PMC7372527; doi:10.12688/hrbopenres.13041.3)
Supplement: Peer review history

# Peer review: Reviewer response for version 2

**DOI:** 10.21956/hrbopenres.14342.r28560

**Published:** 2021-01-20

**Authors:** Katie A. Weatherson

**Affiliations:** 1 Faculty of Medicine, University of British Columbia, Vancouver, BC, Canada

Thank you for your thoughtful consideration of the first review. You have satisfactorily addressed my previous comments. I have some minor additional comments for your consideration:

1. Abstract - last sentence - adaptation or adoption?
2. Analysis - Step 1: Data Extraction section - You still do not describe how you will identify and extract barriers and facilitators from included articles. E.g. How will you identify a 'data point'? If qualitative, how much text will you extract? You seem to include extraction details in Step 2. I would suggest parsing out if in fact two separate steps.
3. General grammatical errors throughout (spelling, tense, incorrect use of semicolons/colons). I would suggest reading thoroughly to identify mistakes.

Is the study design appropriate for the research question?

Yes

Is the rationale for, and objectives of, the study clearly described?

Yes

Are sufficient details of the methods provided to allow replication by others?

Yes

Are the datasets clearly presented in a useable and accessible format?

Not applicable

Reviewer Expertise:

My research primarily focuses on the implementation and effectiveness evaluation of initiatives aiming to improve the health of populations. Specifically related is a review of factors influencing the implementation of physical activity policies in schools.

I confirm that I have read this submission and believe that I have an appropriate level of expertise to confirm that it is of an acceptable scientific standard.

# Peer review: Reviewer response for version 1

**DOI:** 10.21956/hrbopenres.14137.r27547

**Published:** 2020-07-20

**Authors:** Sze Lin Yoong

**Affiliations:** 1 School of Medicine and Public Health, University of Newcastle, Callaghan, NSW, Australia

Thank you for the opportunity to review this protocol. It is clear and well-conducted.

 I have provided some minor comments below for the authors to consider:

1. In the introduction, it may be worth including information on the lack of implementation of food-based guidelines routinely to highlight the challenges with implementing these guidelines and thus enforce the rationale for this review.
2. Some of the rationales for using the TDF may not be relevant to the study. For example, you say that using the TDF will ensure a range of factors examined, but this is only relevant if it is examined in the primary study. Perhaps it is sufficient here to say that using the TDF to synthesis findings provides you with a way of identifying theoretical constructs to target in the development of interventions.
3. The review has two aims – the second aim is not clearly addressed in the methods and synthesis. Are you planning to do this in a subgroup analysis?
4. Repopulation – will you include vocational/technical schools (post-secondary schools) or is this excluded?
5. You say that studies including children’s perceived barriers will be excluded – is this because these are demand focused interventions. Or that children are not involved in implementing food standards? Please provide a rationale.
6. Can you provide a definition or specific example of what constitutes a food standard?
7. The search string looks reasonable although I’m not sure you need a third category (for school only) if your search #2 includes school in most of the search terms. Will you be exploring your mesh headings?
8. Can you provide more information about coding and extraction according to the TDF and how that will be done?
9. Can you add how the second aim will be analysed/explore? Are you exploring barriers by different subgroups? Are you describing your finding narratively or in tabular form?

Is the study design appropriate for the research question?

Yes

Is the rationale for, and objectives of, the study clearly described?

Yes

Are sufficient details of the methods provided to allow replication by others?

Partly

Are the datasets clearly presented in a useable and accessible format?

Not applicable

Reviewer Expertise:

Implementation science, child nutrition, systematic review

I confirm that I have read this submission and believe that I have an appropriate level of expertise to confirm that it is of an acceptable scientific standard.

# Response

**Published:** 2020-12-07

**Authors:** Breda O'Mahony

**Affiliations:** National University of Ireland, Galway, Ireland

Dear Dr. Yoong,

 Thank you for agreeing to be part of the peer review process for this manuscript. We are grateful for all your comments and suggestions on our manuscript. We have carefully considered the comments and have responded to each comment below.

 We hope that our response has provided some clarity which will ultimately improve the quality of the manuscript.

 Yours Sincerely,

 Breda O’ Mahony

**Comment 1:** Thank you for the opportunity to review this protocol. It is clear and well-conducted.

 I have provided some minor comments below for the authors to consider.

***Response 1:*** *We are grateful to the reviewer for their positive comments in relation to the approach for this systematic review. We also appreciate the time that the reviewer gave regarding suggestions and amendments. We hope that we have answered these comments with sufficient detail.*

**Comment 2:** In the introduction, it may be worth including information on the lack of implementation of food-based guidelines routinely to highlight the challenges with implementing these guidelines and thus enforce the rationale for this review.

**Response 2:** *This is a valid suggestion. We have revised paragraph five in the ‘background’ section to include this suggestion.*

 It has been noted that more support must be provided to schools to allow implementation of nutritional guidelines (Abery and Drummond, 2014). Data from Gregoric and colleagues (2015) found that the implementation of school nutrition guidelines were achieved differently at distinct levels.

 Reference:

 Abery E, and Drummond, C. (2014) "Implementation of Mandatory Nutritional Guidelines in South Australian Primary School Canteens: A Qualitative Study." *Improving Schools* 17.1: 41-53.

**Comment 3:** Some of the rationales for using the TDF may not be relevant to the study. For example, you say that using the TDF will ensure a range of factors examined, but this is only relevant if it is examined in the primary study. Perhaps it is sufficient here to say that using the TDF to synthesis findings provides you with a way of identifying theoretical constructs to target in the development of interventions.

**Response 4:** *Thank you for highlighting this. We have implemented your feedback in the updated manuscript to include:*  ‘Using the TDF to synthesize findings provides a mechanism to identify theoretical constructs to target in the development of food standards/guideline implementation’.

**Comment 4:** The review has two aims – the second aim is not clearly addressed in the methods and synthesis. Are you planning to do this in a subgroup analysis?

**Response 4:** *We did not explicitly commit to this comparison as it is dependent on the number of studies retrieved. To allow comparison, subgroup analysis will be performed. This will be completed post hoc, so as to allow the authors to assess if this is an achievable aim. Work by* *Oxman and Guyatt* *(1992)* *and Yusuf et al.* *(1991) will be used to guide subgroup analyses. Our approach to this is included under response 9.*

 References:

 Oxman AD, Guyatt GH. A consumer's guide to subgroup analyses. *Ann Intern Med*. 1992;116(1):78-84. doi:10.7326/0003-4819-116-1-78

 Yusuf S, Wittes J, Probstfield J, Tyroler HA. Analysis and Interpretation of Treatment Effects in Subgroups of Patients in Randomized Clinical Trials. *JAMA.* 1991;266(1):93–98. doi:10.1001/jama.1991.03470010097038

**Comment 5**: Re population – will you include vocational/technical schools (post-secondary schools) or is this excluded?

**Response 5:** *All variations of primary and post primary school will be included. This includes, but is not limited to: high school, junior school, middle school, public school. Pre-primary and post-secondary schools will be excluded.*

*This will be added to the updated manuscript in the ‘population’ section.*

**Comment 6:** You say that studies including children’s perceived barriers will be excluded – is this because these are demand focused interventions. Or that children are not involved in implementing food standards? Please provide a rationale.

***Response 6:*** *As you noted, children are not included because they are demand-side stakeholder.  This systematic review is focused specifically on supply side stakeholders rather than demand-side stakeholders. However, we acknowledge that student’s perceptions and experiences are key, as they are at the core of the standards. Upon completion of this systematic review, the authors intend to undertake research on student’s experiences of the food standards.*

**Comment 7:** Can you provide a definition or specific example of what constitutes a food standard?

***Response 7:*** *Nutrient standards are based on limits and promotions of various nutrients whereas food based standards set requirements on what food can and cannot be served and how frequently (Haroun et al., 2011).*

*For clarity, the terms food and nutrient standard will be described in the revised manuscript (Paragraph 3).*

 Reference:

 Haroun, Dalia & Wood, Lesley & Harper, Clare & Nelson, Michael. (2011). Nutrient-based standards for school lunches complement food-based standards and improve pupils' nutrient intake profile. The British journal of nutrition. 106. 472-4. 10.1017/S0007114511002297.

**Comment 8:** The search string looks reasonable although I’m not sure you need a third category (for school only) if your search #2 includes school in most of the search terms. Will you be exploring your mesh headings?

***Response 8:*** Author one has been trialling various databases searches and looking at other reviews in this area to inform the search string. The addition of education* along with school* has been trialled. To date, this pilot search have produced positive results. MeSH headings are included on the applicable databases.

**Comment 9:** Can you provide more information about coding and extraction according to the TDF and how that will be done?

**Response 9:** *Based on other reviewer comments, we have re-worked the section on data extraction and data synthesis so we avoid repetition but still ensure our comprehensive approach is clear. Further details in relation to coding and extraction are included in the following paragraphs.*

**Data extraction and analysis**

 For data extraction and synthesis, we have adopted a similar approach undertaken in a study protocol by Graham-Rowe et al. (2016). Data will be extracted from all full text studies that meet the inclusion criteria, using a data extraction form. The data extraction form will be created using Microsoft Word. Data to be extracted will include, but is not limited to, the following: Key study information which will include study title, name of the first author, year of publication, country of study, language, study type (qualitative, quantitative and mixed methods studies), study design (e.g. cross sectional and observational), intervention type (e.g. food standards, nutrient standards), type of implementation  (voluntary or mandatory participation), educational setting (primary, post primary, academies etc.) and participant characteristics (canteen staff, head teacher, contracted catering suppliers, food service directors and managers, programme coordinators etc.), sample size, data collection and analysis methods. Data on intervention effects/outcomes, such as change in children’s dietary habits will not form part of this review. A coding manual with the definitions outlined by Cane et al, for the 14 theoretical domains from the TDF, will also be prepared. 

**Analysis**

 Step 1: Data extraction.

 Two review authors, not blind to author or journal information will independently extract from all the full text studies that fulfil the inclusion criteria, using a data extraction form. Each data point will be categorised as either (a) raw data (e.g. quotes from qualitative studies); (b) analysed data from results sections (e.g. statistical analysis from surveys and questionnaires (c) interpretative descriptions and summaries of results from published reports.

**Deductive analysis**

 Step 2: Coding of extracted data

1. Pilot of the coding of data

To ensure validity and reliability, a pilot be will undertaken to allow the review authors to practice coding extracted data into the TDF domains. This will enable comparison of data coding and implement modification, if required. This will be jointly undertaken by the two review authors using the domain definition manual.  Any discrepancies will be resolved by the review team, and if necessary by a third reviewer.

1. Coding extracted data to the TDF

The lead author will continue to code the extracted data, using the domain definition manual, to the TDF domain that they judge to best represent the factor. For qualitative studies, both the theme and the original reporting format of the barrier or facilitator will be extracted. This will include participant quotes, excerpts, quotations or entire passages from studies using documentary analysis, and narrative descriptive summaries of results. If a reported barrier or facilitator is judged to represent more than one domain, it will be coded multiply into its associated domains. Where a factor is not recorded as a barrier or facilitator, we will revert back to the definitions, as previously stated. For quantitative studies, the frequency of all  the barriers and facilitators will be extracted and placed in the correct TDF construct. ****This is important so as to be able to highlight the pertinent factors affecting implementation and provide relevant recommendations. Where mixed method studies are included, we envisage that two data extractions will be required. This ensures all factors will be documented. To avoid overlap, data will be extracted for the qualitative data sheet and for the quantitative data sheet. ****To access coding reliability, a second review author will assess a random sample of 20%.Kappa statistics will be used again to assess inter-rater reliability, with values of 0.75 and higher representing high agreement.

**Inductive analysis**

 Step 3: Thematic synthesis

 Inductive thematic synthesis, based on methods by Thomas and Harden (2008) will also be used to code any data that does not fit into the TDF. This will require a three step process. Step one involves line by line coding of the data that was not coded to the TDF. The second step involves organisation or grouping of these code into associated areas to construct descriptive themes. In step three, the descriptive themes will be compared to refine the relationship between them so as to generate analytical themes.

**Sensitivity analysis**

 Following the synthesis stage, the first author will perform a sensitivity analysis. This will determine if the review findings are sensitive to the following variables: study quality, study methodology (qualitative, quantitative or mixed methods) and location. Both bias and sensitivity reviews will help ensure quality, rigour and transparency (Langer et al, 2015).

**Importance criterion**

 To determine which domains are likely to be the most important factors influencing implementation, each domain in step two will be reviewed. In this review, the frequency of each domain will be examined. Similar to a study protocol by Graham-Rowe et al. (2016)., for qualitative studies we will then consider ‘expressed importance’ within each domain by looking for a statement(s) from the authors interpretation of the study findings articulating what beliefs were reported to be important by the study participants. We acknowledge that this is not a precise process but it has a good fit with the qualitative approach as the meaning, interpretation and prioritisation of the data will be by the authors who have closer familiarity with the primary data, than will be possible by the review team. This process will require discussion and critical judgement by the research team. This will allow the review team, to interpret the domains that have the highest frequency and ‘expressed importance’ as the most important factors in the implementation of school meals standards/guidelines.

 If there is sufficient data, we will explore whether the domains identified as important vary according to the educational establishment i.e. primary and post primary schools. This will be performed by subgroup analysis. Findings will be described in tabular format.

 References:

 Graham-Rowe, E., et al. (2016). "Barriers and enablers to diabetic retinopathy screening attendance: Protocol for a systematic review." Systematic Reviews **5**(1): 134.

 Thomas J, Harden A: Methods for the thematic synthesis of qualitative research in systematic reviews. *BMC Med Res Methodol.* 2008; 8: 45. 

 Langer L, Stewart R, Winters N. Sensitivity analysis in systematic reviews of qualitative evidence: an example from a mixed-methods systematic review . In: Filtering the information overload for better decisions. Abstracts of the 23rd Cochrane Colloquium; 2015 3-7 Oct; Vienna, Austria. John Wiley & Sons; 2015.

**Comment 10:** Can you add how the second aim will be analysed/explore? Are you exploring barriers by different subgroups? Are you describing your finding narratively or in tabular form?

***Response 10:*** *This is now included under response 9 and in the section ‘data extraction and synthesis’.*

 If there are sufficient data, we will explore whether the domains identified as important vary according to the educational establishment i.e. primary and post primary schools. Additionally, if there are sufficient data to compare views across the different supply side stakeholders, this will also be explored. Findings will be described in tabular format.

# Peer review: Reviewer response for version 1

**DOI:** 10.21956/hrbopenres.14137.r27563

**Published:** 2020-07-13

**Authors:** Katie A. Weatherson

**Affiliations:** 1 Faculty of Medicine, University of British Columbia, Vancouver, BC, Canada

The review will address an important question- how to improve the implementation of food and nutrition standards/policies in schools. In general, the study protocol is clearly written and the use of the TDF to categorize barriers and facilitators is a useful addition. I have the following points to clarify the study and overall methodology:

**Abstract:**

  

- Keywords: suggest adding ‘nutrition’

 

**Background:**

- Clarify “their”. E.g., Children, youth.
- Be consistent with use of term “school-based” and “supply-side”. In some places you do not use hyphens.
- Paragraph 3 – This section would benefit from a few concrete details of existing nutrition or food standards for school food (meals/snacks). I suggest adding a couple of examples.
- Add citation: “…difficulties associated with preparing and serving fresh food at school;”
- Paragraph 6 – suggest adding definition of ‘implementation’.
- Add citation : “…later validated in 2012 (version 2 (v2)).”

 

**Methods:** 

- Inclusion criteria – does this include standards for snacks sold in vending machines at school or breakfast programs?
- “The findings will include the following: (i) verbatim quotations from research participants; (ii) excerpts, quotations or entire passages from studies” – change to “may include”
- Outcome sub-section, last sentence: this is a secondary outcome of your review, not of the studies you are including in your review. Revise.
- “The lead author will screen study titles and abstracts to decide whether the full text manuscript should be retrieved. For screening reliability, a second review author will assess a random sample of 20%. Kappa statistics will be used to assess inter-rater reliability, with values of 0.75 and higher representing high agreement.” What will happen in cases of disagreement? Full article will be screened?
- Data extraction sub-section: Revise. You will be extracting barriers and facilitators, NOT a coding manual.  “Data to be extracted will include, … (b) a coding manual with a definition for each of the 14 TDF constructs…”.
- Also, revise “study design (education setting/school type).” Education setting/school type does not reflect study design. Based on your secondary outcome, you should be extracting Education setting (e.g., primary or post primary) data.
- Please provide a rationale for why barriers and facilitators will be extracted from both the results and the discussion sections of included articles. It seems a more valid approach would be to only extract data from the results section, as the discussion section is where authors compare their findings in light of other literature. Extracting information from the discussion would likely lead to double extraction of the same barrier/facilitator (and thus greater emphasis placed on these factors), and/or errors in extraction (extracting results from other comparative studies).
- I also suggest adding more specific details about extraction for qualitative vs quantitative studies. For example, in qualitative studies, results are often presented as themes, with individual quotes used to highlight the theme. Will both the theme and the individual quotes be extracted? In quantitative studies, will all findings be extracted, irrespective of how many participants agreed/reported that the barrier/facilitator existed (i.e., frequency)? I see now that you have reported this under data synthesis – however, I suggest adding to data extraction sub-section. It is important to think through how extracted data will be quantified in your review.
- Quality assessment sub-section: the last sentence does not make sense.
- Data synthesis sub-section: I recommend adding specific mention of the coding manual in this section. Will only one author code the extracted barriers/facilitators to the TDF domains?

 

**Discussion:**

  

- Paragraph 3 – Add sentence about specific evidence gap before referring to it in sentence two.

Is the study design appropriate for the research question?

Yes

Is the rationale for, and objectives of, the study clearly described?

Yes

Are sufficient details of the methods provided to allow replication by others?

Yes

Are the datasets clearly presented in a useable and accessible format?

Not applicable

Reviewer Expertise:

My research primarily focuses on the implementation and effectiveness evaluation of initiatives aiming to improve the health of populations. Specifically related is a review of factors influencing the implementation of physical activity policies in schools.

I confirm that I have read this submission and believe that I have an appropriate level of expertise to confirm that it is of an acceptable scientific standard, however I have significant reservations, as outlined above.

# Response

**Published:** 2020-12-07

**Authors:** Breda O'Mahony

**Affiliations:** National University of Ireland, Galway, Ireland

Dear Dr. Weatherson,

 Thank you for agreeing to be part of the peer review process for this manuscript. We are grateful for all your comments and suggestions on our manuscript. We have carefully considered the comments and have responded to each comment below.

 We hope that our response has provided come clarity which will ultimately improve the quality of the manuscript.

 Yours Sincerely,

 Breda O’ Mahony

**Comment 1:** The review will address an important question- how to improve the implementation of food and nutrition standards/policies in schools. In general, the study protocol is clearly written and the use of the TDF to categorize barriers and facilitators is a useful addition. I have the following points to clarify the study and overall methodology:

***Response 1:*** *We are grateful to the reviewer for their positive comments in relation to the concept and rationale for this systematic review. We also appreciate the time that the reviewer gave regarding suggestions and amendments.*

**Abstract**

**Comment 2:** Abstract: Keywords: suggest adding ‘nutrition’

***Response 2:*** *HRB Open guidelines state that up to eight keywords can be used. We have removed the key word ‘schools’ and replaced it with ‘nutrition’. We made this swap as school is represented in keywords ‘school meal standards’ and ‘school food guidelines’.*

**Background**

**Comment 3:** Clarify “their”. E.g., Children, youth.

***Response 3:*** *We have removed the word ‘their’ and replaced this with ‘child and adolescent’. Paragraph 1 now reads: Schools are a key setting for the promotion of health and well-being* *^1^* *^–^* *^3^* *. They are one of the most effective ways of reaching a large segment of the population* *^4^* *^,^* *^5^* *, with no other institution having as much continuous contact and influence during the first stages of a **child **life* *^6^* *.*

**Comment 4:** Be consistent with use of term “school-based” and “supply-side”. In some places you do not use hyphens.

***Response 4:*** *Thank you for pointing out this. We will ensure that the use of hyphens is consistent in the updated version (version 2).*

**Comment 5:** Paragraph 3 – This section would benefit from a few concrete details of existing nutrition or food standards for school food (meals/snacks). I suggest adding a couple of examples.

**Response 5:** *Thank you for this suggestion. This paragraph has now been extended to:*

*To date, a number of countries and regions around the world have introduced nutrition or food standards for school food on a mandatory basis. These include Finland in 1943, Sweden in 1997 , Norway in 2001, a reintroduction of compulsory guidelines after 21 years in England in 2001, Slovenia in 2010, and an updated National School Lunch Programme in United States of America (USA) in 2012, which will be phased into all schools by 2023. Nutrient standards are based on limits and promotions of various nutrients whereas food based standards set requirements on what food can and cannot be served and how frequently (Haroun et al., 2011).  Examples of standards include the Irish Nutrition Standards for School meals (Department of Health, 2017). The food based standards set out requirements for each meal type e.g. for breakfast a minimum of two items should be provided, one serving of wholemeal or wholegrain cereals and breads, and one serving of either milk, yoghurt or cheese or fruit.*

**Comment 6:** Add citation: “…difficulties associated with preparing and serving fresh food at school;”

***Response 6:*** *Citation has been added and will be included to the updated version.  It will read* “…difficulties associated with preparing and serving fresh food at school **^20^**;”

 20. Tabak RG, Moreland-Russell S: Food Service Perspectives on National School Lunch Program Implementation. *Health Behav and Policy Rev.* 2015; **2**(5): 362–371

**Comment 7:** Paragraph 6 – suggest adding definition of ‘implementation’.

**Response7:** *Thank you for this suggestion, we added an explanation of ‘implementation’ to paragraph 5. This will be added to the updated version and paragraph 5 will now read:*

 It has been noted that more support must be provided to schools to allow implementation of nutritional guidelines (Abery and Drummond, 2014). Data from Gregoric and colleagues (2015) ^13^ found that the implementation of school nutrition guidelines differed by schools within the same region. **Implementation as a process and a science is complex to study because of the numerous factors affecting implementation, its process, results and potential solutions (Peters et al, 2014).**

 Reference:

 Peters DH, Adam T, Alonge O, Agyepong IA, Tran N. (2014) "Republished research: Implementation research: what it is and how to do it." British Journal of Sports Medicine **48**(8): 731.

**Comment 8:** Add citation : “…later validated in 2012 (version 2 (v2)).”

**Response 8:** *Thank you for highlighting this omission; a citation has now been added* “…later validated in 2012 (version 2 (v2)) **^36^**.”

 Reference:

 36. Atkins, L., Francis, J., Islam, R. *et al.* A guide to using the Theoretical Domains Framework of behaviour change to investigate implementation problems. *Implementation Sci* **12, **77 (2017)

**Methods**

**Comment 9:** Inclusion criteria – does this include standards for snacks sold in vending machines at school or breakfast programs?

***Response 9:*** *Yes, this includes standards for snacks sold in vending machines at school and or school breakfast programs. This will be made more explicit in the updated manuscript, under paragraph ‘Intervention’.*

**Comment 10:** “The findings will include the following: (i) verbatim quotations from research participants; (ii) excerpts, quotations or entire passages from studies” – change to “may include”

***Response 10:*** *This change will be made to the updated version and will read *“The findings **may** include the following: (i) verbatim quotations from research participants; (ii) excerpts, quotations or entire passages from studies”.

**Comment 11:** Outcome sub-section, last sentence: this is a secondary outcome of your review, not of the studies you are including in your review. Revise

***Response 11:*** *This will now be revised to:*

 A secondary outcome **of the review **may include comparing the barriers and facilitators between a primary and post primary school setting.

**Comment 12:** “The lead author will screen study titles and abstracts to decide whether the full text manuscript should be retrieved. For screening reliability, a second review author will assess a random sample of 20%. Kappa statistics will be used to assess inter-rater reliability, with values of 0.75 and higher representing high agreement.” What will happen in cases of disagreement? Full article will be screened?

***Response 12:*** *In the case of disagreement, full article will be screened. This will be added to the revised manuscript.*

**Comment 13:** Data extraction sub-section: Revise. You will be extracting barriers and facilitators, NOT a coding manual.  “Data to be extracted will include, … (b) a coding manual with a definition for each of the 14 TDF constructs…”.

**Response 13:** *Thank you for noticing this error. This has now been removed and added instead to the data extraction and synthesis paragraph.*

**Comment 14:** Also, revise “study design (education setting/school type).” Education setting/school type does not reflect study design. Based on your secondary outcome, you should be extracting Education setting (e.g., primary or post primary) data.

***Response 14:*** *Apologies for the error. The text will be amended to education setting (e.g. primary or post primary) in the updated manuscript.*

**Comment 15:** Please provide a rationale for why barriers and facilitators will be extracted from both the results and the discussion sections of included articles. It seems a more valid approach would be to only extract data from the results section, as the discussion section is where authors compare their findings in light of other literature. Extracting information from the discussion would likely lead to double extraction of the same barrier/facilitator (and thus greater emphasis placed on these factors), and/or errors in extraction (extracting results from other comparative studies).

**Response 15:** *The decision to include the discussion sections (with the results sections) is for qualitative studies only. Some journals have limited word counts so the inclusion of author interpretations can ensure findings are not omitted. Thus, the inclusion of author’s interpretations in qualitative studies allows for a more in-depth syntheses.*

*This will be more explicit in the updated manuscript.*

**Comment 16: **I also suggest adding more specific details about extraction for qualitative vs quantitative studies. For example, in qualitative studies, results are often presented as themes, with individual quotes used to highlight the theme. Will both the theme and the individual quotes be extracted? In quantitative studies, will all findings be extracted, irrespective of how many participants agreed/reported that the barrier/facilitator existed (i.e., frequency)? I see now that you have reported this under data synthesis – however, I suggest adding to data extraction sub-section. It is important to think through how extracted data will be quantified in your review.

***Response 16:*** *We have taken your comment on board and we have revised the data extraction paragraph to take on board all of the reviewers’ comments and suggestions. This will now read:*

**Data extraction and analysis materials/tools**

 For data extraction and synthesis, we have adopted a similar approach undertaken in a study protocol by Graham-Rowe et al. (2016). Data will be extracted from all full text studies that meet the inclusion criteria, using a data extraction form. The data extraction form will be created using Microsoft Word. Data to be extracted will include, but is not limited to, the following: Key study information which will include study title, name of the first author, year of publication, country of study, language, study type (qualitative, quantitative and mixed methods studies), study design (e.g. cross sectional and observational), intervention type (e.g. food standards, nutrient standards), type of implementation  (voluntary or mandatory participation), educational setting (primary, post primary, academies etc.) and participant characteristics (canteen staff, head teacher, contracted catering suppliers, food service directors and managers, programme coordinators etc.), sample size, data collection and analysis methods. Data on intervention effects/outcomes, such as change in children’s dietary habits will not form part of this review. A coding manual with the definitions outlined by Cane et al, for the 14 theoretical domains from the TDF, will also be prepared. 

**Analysis**

 Step 1: Data extraction.

 Two review authors, not blind to author or journal information will independently extract from all the full text studies that fulfil the inclusion criteria, using a data extraction form. Each data point will be categorised as either (a) raw data (e.g. quotes from qualitative studies); (b) analysed data from results sections (e.g. statistical analysis from surveys and questionnaires (c) interpretative descriptions and summaries of results from published reports.

**Deductive analysis**

 Step 2: Coding of extracted data

1. Pilot of the coding of data

To ensure validity and reliability, a pilot be will undertaken to allow the review authors to practice coding extracted data into the TDF domains. This will enable comparison of data coding and implement modification, if required. This will be jointly undertaken by the two review authors using the domain definition manual.  Any discrepancies will be resolved by the review team, and if necessary by a third reviewer.

1. Coding extracted data to the TDF

The lead author will continue to code the extracted data, using the domain definition manual, to the TDF domain that they judge to best represent the factor. For qualitative studies, both the theme and the original reporting format of the barrier or facilitator will be extracted. This will include participant quotes, excerpts, quotations or entire passages from studies using documentary analysis, and narrative descriptive summaries of results. If a reported barrier or facilitator is judged to represent more than one domain, it will be coded multiply into its associated domains. Where a factor is not recorded as a barrier or facilitator, we will revert back to the definitions, as previously stated. For quantitative studies, the frequency of all  the barriers and facilitators will be extracted and placed in the correct TDF construct. ****This is important so as to be able to highlight the pertinent factors affecting implementation and provide relevant recommendations. Where mixed method studies are included, we envisage that two data extractions will be required. This ensures all factors will be documented. To avoid overlap, data will be extracted for the qualitative data sheet and for the quantitative data sheet. ****To access coding reliability, a second review author will assess a random sample of 20%.Kappa statistics will be used again to assess inter-rater reliability, with values of 0.75 and higher representing high agreement.

**Inductive analysis**

 Step 3: Thematic synthesis

 Inductive thematic synthesis, based on methods by Thomas and Harden (2008) will also be used to code any data that does not fit into the TDF. This will require a three step process. Step one involves line by line coding of the data that was not coded to the TDF. The second step involves organisation or grouping of these code into associated areas to construct descriptive themes. In step three, the descriptive themes will be compared to refine the relationship between them so as to generate analytical themes.

**Sensitivity analysis**

 Following the synthesis stage, the first author will perform a sensitivity analysis. This will determine if the review findings are sensitive to the following variables: study quality, study methodology (qualitative, quantitative or mixed methods), type of implementation (voluntary or mandatory participation) and location. Both bias and sensitivity reviews will help ensure quality, rigour and transparency (Langer et al, 2015)

**Importance criterion**

 To determine which domains are likely to be the most important factors influencing implementation, each domain in step two will be reviewed. In this review, the frequency of each domain will be examined. Similar to a study protocol by Graham-Rowe et al. (2016)., for qualitative studies we will then consider ‘expressed importance’ within each domain by looking for a statement(s) from the authors interpretation of the study findings articulating what beliefs were reported to be important by the study participants. We acknowledge that this is not a precise process but it has a good fit with the qualitative approach as the meaning, interpretation and prioritisation of the data will be by the authors who have closer familiarity with the primary data, than will be possible by the review team. This process will require discussion and critical judgement by the research team. This will allow the review team, to interpret the domains that have the highest frequency and ‘expressed importance’ as the most important factors in the implementation of school meals standards/guidelines.

 If there is sufficient data, we will explore whether the domains identified as important vary according to the educational establishment i.e. primary and post primary schools. This will be performed by subgroup analysis. Findings will be described in tabular format.

 References:

 Graham-Rowe, E., et al. (2016). "Barriers and enablers to diabetic retinopathy screening attendance: Protocol for a systematic review." Systematic Reviews **5**(1): 134.

 Thomas J, Harden A: Methods for the thematic synthesis of qualitative research in systematic reviews. *BMC Med Res Methodol.* 2008; 8: 45. 

 Langer L, Stewart R, Winters N. Sensitivity analysis in systematic reviews of qualitative evidence: an example from a mixed-methods systematic review . In: Filtering the information overload for better decisions. Abstracts of the 23rd Cochrane Colloquium; 2015 3-7 Oct; Vienna, Austria. John Wiley & Sons; 2015.

**Comment 17:** Quality assessment sub-section: the last sentence does not make sense.

***Response 17:*** *This sentence will now read “When disagreements between reviewers cannot be easily resolved, a third independent reviewer will become involved in order to reach consensus.*

**Comment 18:** Data synthesis sub-section: I recommend adding specific mention of the coding manual in this section. Will only one author code the extracted barriers/facilitators to the TDF domains?

***Response 18:*** *We have expanded the information under extraction and analyses. This includes more specific information on the TDF. This paragraph will now read:*

**Deductive analysis**

 Step 2: Coding of extracted data

1. Pilot of the coding of data

To ensure validity and reliability, a pilot be will undertaken to allow the review authors to practice coding extracted data into the TDF domains. This will enable comparison of data coding and implement modification, if required. This will be jointly undertaken by the two review authors using the domain definition manual.  Any discrepancies will be resolved by the review team, and if necessary by a third reviewer.

1. Coding extracted data to the TDF

The lead author will continue to code the extracted data, using the domain definition manual, to the TDF domain that they judge to best represent the factor. For qualitative studies, both the theme and the original reporting format of the barrier or facilitator will be extracted. This will include participant quotes, excerpts, quotations or entire passages from studies using documentary analysis, and narrative descriptive summaries of results. If a reported barrier or facilitator is judged to represent more than one domain, it will be coded multiply into its associated domains. Where a factor is not recorded as a barrier or facilitator, we will revert back to the definitions, as previously stated. For quantitative studies, the frequency of all  the barriers and facilitators will be extracted and placed in the correct TDF construct. ****This is important so as to be able to highlight the pertinent factors affecting implementation and provide relevant recommendations. Where mixed method studies are included, we envisage that two data extractions will be required. This ensures all factors will be documented. To avoid overlap, data will be extracted for the qualitative data sheet and for the quantitative data sheet. ****To access coding reliability, a second review author will assess a random sample of 20%.Kappa statistics will be used again to assess inter-rater reliability, with values of 0.75 and higher representing high agreement.

**Discussion**

**Comment 19:** Paragraph 3 – Add sentence about specific evidence gap before referring to it in sentence two.

***Response 19:*** *Paragraph 3 under the Discussion section will now read-*

**A number of studies have identified various factors that affect implementation, however there has been limited synthesis of this information from a stakeholder’s perspective. Ronto and colleagues (2020) recent systematic review analysed the implementation and/or compliance with school-based healthy food and beverage policies. The eligibility criteria included policy relating to nutrition guidelines, regulations and/or restrictions on food and beverage availability, advertisement, placement or price. There was no exclusion criteria in relation to study participants, but grey literature was not accessed. Our mixed methods review is specific to the implementation of food/nutrient based standards from a stakeholder’s perspective. To ensure a comprehensive review is undertaken, a variety of electronic databases and grey literature sources will be assessed. **Given ****the potential impact that school meal guidelines and standards can have on the health and wellbeing outcomes of children and adolescents, understanding the factors that affect their implementation is key. We are confident that the depth of this review will provide a holistic understanding of the factors as all types of studies; qualitative and quantitative or both, including grey literature, will be accessed. Furthermore, there will no language or publication date restrictions. The review will follow academic rigour and will include a number of strategies for validity, reliability and to reduce the effects of bias. This will be achieved by having clear and detailed inclusion and exclusion criteria, independent reviewers, the use of PRISMA guidelines, a MMAT, and by using computer packages for data and quality management. Finally, where deviations from this protocol occur, this will be justified and discussed in the systematic review upon publication and will be documented on PROSPERO.

# Peer review: Reviewer response for version 1

**DOI:** 10.21956/hrbopenres.14137.r27383

**Published:** 2020-05-21

**Authors:** Suzanne Spence

**Affiliations:** 1 Human Nutrition Research Centre, Population Health Sciences Institute, Faculty of Medical Sciences, Newcastle University, Newcastle upon Tyne, UK

This is an interesting and well thought through concept for a systematic review. I am not an expert in systematic review methodologies and therefore cannot comment in great detail on that aspect. Overall, the rationale is clear, I have a few wider comments for the authors to consider for this review.

**Objectives:**

- The authors have a primary objective to identify and synthesise the existing evidence on the barriers and facilitators to implementing food or nutrition standards for school food from supply-side stakeholders. Would you please clarify the term supply-side stakeholders: does this include school caterers, headteachers, school business managers, and canteen staff too?
- The authors also mention a secondary objective may be included to compare the barriers and facilitators between a primary and post-primary school setting. I think this objective should be included from the outset - primary schools are very different settings to secondary schools/academies and will be an important aspect of the review to reflect this in the analysis and discussions.

 

**Population:**

 The authors are excluding studies that involve school children’s perceived barriers and facilitators – is it possible to give a rationale for excluding children’s perceptions?

**Intervention/Methods:**

 If possible, if the authors could provide some clarification on the following points about the intervention that would be helpful. The authors note they will include studies:

- Where interventions delivered in educational establishments and school food standards have been implemented on a voluntary or mandatory basis.
- No restriction on the type of standard i.e. nutrient and food-based standards will be included
- And the international span of countries

How do the authors intend to explore/take into account the effect of the difference between voluntary and mandatory on barriers/success of implementation – these are quite different approaches to implementation.

 Similarly, food and nutrient-based standards vary globally re requirements/complexity – how will country effect be captured in review? Both complexity of standards and variation by country may well influence the barriers/success of the implementation of standards.

 While there is a clear overview/outcome of data extraction for the qualitative component I am unclear about the outcome for the quantitative aspect, is it an effect on nutrition, children’s diets, etc. A bit more clarification for the quantitative outcome is required.

Is the study design appropriate for the research question?

Yes

Is the rationale for, and objectives of, the study clearly described?

Yes

Are sufficient details of the methods provided to allow replication by others?

Yes

Are the datasets clearly presented in a useable and accessible format?

Not applicable

Reviewer Expertise:

Public Health Nutrition, children's dietary intake, policy evaluation of school food and nutrient standards

I confirm that I have read this submission and believe that I have an appropriate level of expertise to confirm that it is of an acceptable scientific standard, however I have significant reservations, as outlined above.

# Response

**Published:** 2020-06-17

**Authors:** Breda O'Mahony

**Affiliations:** National University of Ireland, Galway, Ireland

Dear Dr. Spence,

Thank you for agreeing to be part of the peer review process for this manuscript. We are grateful for all your comments and suggestions on our manuscript. We have carefully considered the comments and have responded to each comment below.

We hope that our response has provided come clarity which will ultimately improve the quality of the manuscript.

Yours Sincerely,

Breda O’ Mahony

Comment 1

This is an interesting and well thought through concept for a systematic review. I am not an expert in systematic review methodologies and therefore cannot comment in great detail on that aspect. Overall, the rationale is clear, I have a few wider comments for the authors to consider for this review.

Response 1

Thank you for your positive comments in relation to the concept and rationale for this systematic review.

Comment 2

Objective 1: The authors have a primary objective to identify and synthesise the existing evidence on the barriers and facilitators to implementing food or nutrition standards for school food from supply-side stakeholders. Would you please clarify the term supply-side stakeholders: does this include school caterers, headteachers, school business managers, and canteen staff too?

Response 2

The reviewer’s interpretation of supply side stakeholder is correct. The term supply side stakeholders refers, but is not limited to catering management and staff, school principals/managers, contracted catering suppliers, food service directors and managers, programme coordinators (this is outlined under paragraph ‘Population’ on page 4). Studies involving school children’s perceived barriers and facilitators will not be included. Furthermore, standards in pre-schools and third level settings will also be excluded.

Comment 3

Objective 2: The authors also mention a secondary objective may be included to compare the barriers and facilitators between a primary and post-primary school setting. I think this objective should be included from the outset - primary schools are very different settings to secondary schools/academies and will be an important aspect of the review to reflect this in the analysis and discussions.

Response 3

Thank you for this suggestion. We did not explicitly commit to this comparison as it is dependent on the number of studies retrieved. The manuscript will be amended and will read: “A secondary objective is to compare the barriers and facilitators between a primary and post primary school setting”.

Comment 4

Population: The authors are excluding studies that involve school children’s perceived barriers and facilitators – is it possible to give a rationale for excluding children’s perceptions?

Response 4

This is a valuable point, however we believe that this would greatly expand the focus of this systematic review. This systematic review is focused on supply side stakeholders rather than demand-side stakeholders (i.e. students). However, we acknowledge that student’s perceptions and experiences are key, as they are at the core of the standards. Upon completion of this systematic review, the authors intend to undertake research on student’s experiences of the food standards. 

Comment 5

Intervention

If possible, if the authors could provide some clarification on the following points about the intervention that would be helpful. The authors note they will include studies:

Where interventions delivered in educational establishments and school food standards have been implemented on a voluntary or mandatory basis.

Response 5

The authors aim to capture all internationally suitable research that relates to the implementation of school food standards. After carrying out a preliminary scoping search, it illustrated that some schools implemented food standards on a voluntary or mandatory basis, e.g. in response to national or regional policy. The scoping search also identified studies where school food standards were voluntarily implemented for research purposes. Such studies will also be included.

Comment 6

If possible, if the authors could provide some clarification on the following points about the intervention that would be helpful. The authors note they will include studies:

No restriction on the type of standard i.e. nutrient and food-based standards will be included

Response 6

After conducting a preliminary search to inform the protocol and systematic review, we found that some jurisdictions used the term nutrient standards (or similar deviations) whilst other jurisdictions used the term food based standards (or similar deviations). Both terms are relevant as they focus on food provision. Nutrient standards are based on limits and promotions of various nutrients whereas food based standards set requirements on what food can and cannot be served and how frequently (Haroun et al., 2011). To ensure all suitable studies are captured, this has been reflected in the eligibility study criteria for inclusion/exclusion. Our eligibility criteria clearly rules out studies based on dietary guidelines, healthy eating policies etc.

Comment 7

If possible, if the authors could provide some clarification on the following points about the intervention that would be helpful. The authors note they will include studies:

And the international span of countries

Response 7

The authors decided rather than selecting certain jurisdictions, to expand and capture all suitable international research on nutrient or food based standards. For this reason, we decided not to apply an English language restriction.

Comment 8

How do the authors intend to explore/take into account the effect of the difference between voluntary and mandatory on barriers/success of implementation – these are quite different approaches to implementation.

Response 8

Due to similarities in responses between comment 8 & 9, the response to comment 8 will be outlined under response 9.

Comment 9

Similarly, food and nutrient-based standards vary globally re requirements/complexity – how will country effect be captured in review? Both complexity of standards and variation by country may well influence the barriers/success of the implementation of standards.

Response 9 

The author’s acknowledge the implementation is context dependent and have noted to ensure that this forms part of the key information. As part of data extraction and testing of the synthesis, the authors will include the intervention type (i.e. nutrient, food standard) and type of implementation (intervention, voluntary or mandatory participation) and study setting (primary or post primary) as part of data extraction and testing of the synthesis.

The authors also recognise that different types of supply-side stakeholders (i.e. canteen staff, school principal) may report different barriers and facilitators to implementation of menu labeling. We will ensure that this forms part of the testing synthesis.

Key study information will include study title, name of the first author, year of publication, **country of study**, language, study type (qualitative, quantitative and mixed methods studies), intervention type (e.g. food standards, nutrient standards), type of implementation  (voluntary or mandatory participation), study setting (primary, post primary, academies etc.) and participant characteristics (canteen staff, head teacher, contracted catering suppliers, food service directors and managers, programme coordinators etc.), sample size, data collection and analysis methods. Data on intervention effects/outcomes, such as change in children’s dietary habits will not form part of this review. The outcome that forms part of PICOS are the barriers and facilitators to the implementation of nutrition standards for school meals.

Moreover, a sensitivity analysis will be conducted in order to determine if the synthesis is sensitive to the following: study design, quality assessment, intervention type (e.g. food standards, nutrient standards), type of implementation (voluntary or mandatory participation), study setting (primary, post primary, academies etc.) and participant characteristics (canteen staff, head teacher, contracted catering suppliers, food service directors and managers, programme coordinators etc.) and location (e.g. Europe, America, Australia). As barriers and facilitators to implementation are highly context-dependent, the sensitivity analysis will be an important step in assessing if the synthesis is sensitive to contextual factors such as intervention type, implementation type and time of data collection.

Comment 10

While there is a clear overview/outcome of data extraction for the qualitative component I am unclear about the outcome for the quantitative aspect, is it an effect on nutrition, children’s diets, etc. A bit more clarification for the quantitative outcome is required.

Response 10

For quantitative studies the proportion of respondents (study participants) that identified each barrier/facilitator will be extracted, coded and mapped against the Theoretical Domains Framework. Study participants will be listed so as to be able to discern between the various supply side stakeholders. To provide some context on the extent of the barrier/facilitator, the frequency of each factor reported within studies will also be calculated. This information will be added to the manuscript.
